# Genotype by heat conditions interaction effects on growth and litter traits in rabbits

**DOI:** 10.3389/fvets.2022.1018625

**Published:** 2022-11-22

**Authors:** Mohamed Ragab, Ibrhim Elkhaiat, Hassan Younis, Marwa Ahmed, Mostafa Helal

**Affiliations:** ^1^Poultry Production Department, Faculty of Agriculture, Kafrelsheikh University, Kafrelsheikh, Egypt; ^2^Institute for Animal Science and Technology, Universitat Politècnica de València, Camino de Vera S/N, Valencia, Spain; ^3^Department of Animal Production, National Reserach Center, Dokki, Giza, Egypt; ^4^Department of Animal Production, Faculty of Agriculture, Cairo University, Giza, Egypt

**Keywords:** G x E interaction, heat stress, litter size, growth traits, rabbits

## Abstract

Heat stress has severe impacts on rabbit performance because they have difficulty getting rid of excess heat. The interaction between genetic and environmental factors plays a vital role in the adaptation process. The current study aimed to evaluate the effects of interaction between the genotype and heat conditions (G×H) on litter size and growth traits. Two rabbit lines were used in the current study, Egyptian maternal line (APRI) and New Zealand White (NZW). The rabbits were raised under normal (22°C) or heat stress (35°C) conditions. The does were raised in individual cages, and their kits were reared under the same conditions. Negative effects of heat conditions were noted for litter traits, as heat stress had significantly reduced the number of born alive, total born, and marketed number rabbits by about 16, 11, and 25%, respectively. Moreover, growth traits were reduced under heat stress conditions compared to normal temperature conditions. Significant differences between genotypes were observed, APRI rabbits were higher than NWZ rabbits in the total born, number of born alive, number weaned, and the number of marketed rabbits by 10, 8, 11, and 10%, respectively. Genotype by heat conditions interaction effects were observed, APRI litters under normal conditions showed significantly higher litter traits than NWZ litters, whereas relevant reductions in litter traits of APRI line than NZW litters when reared in heat conditions. For growth traits, the differences between the two lines under normal conditions were reduced when animals of the two lines were raised under heat stress, for BW_28_, BW_42_, BW_63_, ADG_28−42_, and DF_28−42_ while these differences were increased for ADG_42−63_, DF_28−42_ and feed conversion during the whole fattening period. Based on the observed G × H interactions, it is important to select the animals under the same production conditions to raise rabbits that can cope with the expected global warming conditions.

## Introduction

Recently, global warming is leading to an increase in the atmospheric temperature by about 0.07°C per year, with predicted increases ranging from 2.6 to 4.7°C by the year 2100 (1). Rabbit is a good source of meat and can contribute to filling the gap between the production and consumption of animal protein. Nevertheless, rabbit is susceptible to heat stress (HS) conditions (2, 3). This change in atmospheric temperature will generate heat stress conditions in tropical and subtropical regions like the south of the Mediterranean, especially in the open production system, which is the most common farm type in Egypt (4). Rabbit is sensitive to high environmental temperatures and has a low capacity to cope with high ambient temperatures (5), which negatively affect health, behavior, physiology, reproduction, welfare, meat quality, and production performance of rabbits (6, 7). Therefore, heat stress is associated with economic losses to the rabbit industry. Moreover, this impact could be measured either directly *via* the performance of the animal, or indirectly through biological stress markers (8, 9).

On the other hand, the responses to heat stress differ between animals of different genetic backgrounds, in which different breeds and lines respond differently to the different environments (10, 11). In general, local or indigenous animals have more capability for adaptation to the changes in local environmental conditions compared with exotic animals, this can be attributed to the genetic compositions that enable them to adapt the local environmental conditions (12, 13). To cope with the rapid changes in the environmental conditions, a better understanding of the role of G × E interaction in environmental adaptation is needed which is one of the main components of robustness. The G × E interaction is observed when the effects of the environmental factors differ from one breed to another, and the presence of G × E interaction may result in the re-ranking of genotypes (14), which makes one breed more sensitive to thermal stress than other. Moreover, in cattle, Misztal et al. (15) indicated that the selection for milk production, without considering the HS, will produce animals more susceptible to HS. With the desire for lines for robustness in the rabbit industry, an Egyptian Hispanic project developed a long and extensive genetic research program to develop new lines of interest for both extensive and intensive rabbit production. During this project, the Spanish maternal line (Line V), which is adapted to hot conditions, was crossed with local Egyptian rabbit breeds to produce lines that maintain a reasonable productive capacity and adaptation to heat. By this project, three rabbit lines were founded called Alexandria, Moshtohor, and APRI (16–18).

In rabbits, reproductive performance of does is important, and litter traits are the most important traits, which affect the economic benefits of rabbit production that provide the needed animal protein with low capital outlay and time (7). In this way, Cartuche et al. (19) reported that feed conversion rate during the fattening period, litter size, weaning survival, fattening survival and daily gain during the fattening period are the most economic traits in rabbits. These traits are severely affected when rabbits are subjected to heat stress conditions. Therefore, the purpose of this study was to compare the genetic response of Egyptian maternal line and exotic rabbit breed under normal and heat stress conditions.

## Materials and methods

The study was carried out at the experimental rabbit farm of the Faculty of Agriculture, Kafrelsheikh University (Kafr Elsheikh, Egypt). This study was conducted after the approval of the Animal Care and Ethics Committee of Kafrelsheikh University (approval number, KFS1345/10) and also approved by IACUC at Cairo University (CU/II/F/19/19).

### Animals and management

The animals used in the current study belonged to line APRI and New Zealand White breed (exotic breeds raised at the commercial level in Egypt many years ago). APRI line is an Egyptian maternal line developed in 2008, by crossing bucks of Baladi Red rabbits (a local breed) with does of V line rabbits (18). After its foundation, the generated rabbits were then selected for weaning weight (20).

The first mating of the does was done around 18 weeks of age. The does were then served 10–12 days post kindling by its assigned buck. On day 12 post-mating, the does were palpated to detect pregnancy, and non-pregnant does were returned to a new mating. The mating system was designed to avoid inbreeding. The maximum number of weekly services allowed to a buck was two. Five days before the expected day for kindling, the nest boxes were prepared. Litters born were examined and recorded for total born and number born alive. The litter born stayed with their mothers, without fostering, for about 35 days the young rabbits were weaned and then individually identified by tattooed number in the ear.

All rabbits were fed *ad libitum* commercial pelleted diet (17.5% crude protein, 15%−16% crude fiber, 2.5% either extract, 0.6% minerals mixture, 67.4% soluble carbohydrates, and 2300:2500 kcal/kg diet). Water was also provided *ad libitum* from nipple drinkers. The rabbits were housed in cages, where 100 cages were distributed equally for each rabbit line. Sixty cages were used to raise does under the normal environmental temperatures (NC), with an average of 18–26°C. Whereas, the heat-stress room included 40 cages, to keep the does under heat conditions (HC, 33–37°C). The heat conditions were applied for 7 h daily from 9:00 to 16:00, where it peaked daily temperatures during the summer season in Egypt, followed by the daily temperature cycle.

At first mating age, 100 does were used in this experiment, females were distributed almost the same between the two genetic groups, and animals were housed in individual cages. When one of the does died or was culled, it was replaced by another doe of the same genetic line. After weaning, during the fattening period (i.e., from 28 to 63 days of age), the kits produced under each environmental condition were grown under the same condition as their mothers. All weaned rabbits (2037 rabbits) were housed in collective cages (50 × 60 × 30 cm) of six animals in the same environmental conditions (normal or heat stress) until they reached the marketing age (around 63 days).

### Traits and statistical analyses

The considered traits were litter traits including total born (TB), number born alive (NBA), number weaned (NW), number marketed (NM), in addition to growth traits including individual body weights [at weaning (BW_28_), at 42 days of age (BW_42_), at marketing age (BW_63_)], average daily weight gain [from weaning to marketing (ADG_28−63_), from weaning to 42 days of age (ADG_28−42_), from 42days of age to marketing (ADG_42−63_)], daily feed intake (calculated by dividing total feed consumption by the number of animals in each cage, and to avoid the biased calculation for feed intake, if an animal died in a cage during any week, the feed intake was calculated immediately including the dead and live animals) was calculated from weaning to marketing (DF_28−63_), from weaning to 42 days of age (DF_28−42_), from 42 days of age to marketing (DF_42−63_), and feed conversion ratio during the fattening period (FC_28− 63_).

All the obtained data were subjected to analysis, the univariate mixed model was fitted to a total of 504 parities for APRI and NZW were analyzed.

Litter traits were analyzed using the following univariate mixed model:


$$\begin{array}{l} Y_{ijkl}\;=\;LRYS_{i}\;+\;PS_{j}\;+\;p_{k}\;+\;e_{ijkl,} \end{array}$$

where *Y*_*ijkl*_ is the record *l*^*th*^ of litter traits being analyzed (TB, NBA, NW and NM), corresponding to the *k*^*th*^ doe which was in the physiological status *j*^*th*^ and belongs to the line-room-year-season combination *i*^*th*^; *LRYS*_*i*_ is the fixed effect of the line-room-year-season combination [combination of each line by room by year season (every 3 months): 10 levels]; *PS*_*j*_ is the fixed effect of the doe physiological state (three levels depending on the parity order and lactation state at mating, one is for nulliparous does, two for primiparous, and three for multiparous lactating); *p*_*k*_ is the random permanent environmental plus non-additive genetic effects of the doe; and *e*_*ijkl*_ is the random residual of the model.

The model for growth traits was:


$$\begin{array}{l} Y_{ijkl}=LRYS_{i}+OP_{j}+\beta {\left(NBA\right)}_{k}+lo_{l}+e_{ijkl}\; \end{array}$$

where: *Y*_*ijkl*_ is the record of the growth trait of animal *l*; *LRYS*_*i*_ is the effect of line-room-year-season combination, line of animal *l* and the year-season of parity (one year-season every 3 months: nine levels for all lines); *OP*_*j*_ is the effect of the order of parity (five levels: 1^st^, 2^nd^, and >2^nd^), *NBA*_*k*_ is the number of born alive in the litter in which the young rabbit was born and β is the regression coefficient on this covariate; *lo*_*k*_ is the random effect of the litter in which the animal was born and *e*_*ijkl*_ is the residual effect. Sex effect was not included because sexual dimorphism in rabbits is thought to either not exist or to arise only late in life (21). The prior distribution for the permanent environmental effect (**p**) and litter effect (**lo**) were $\text{p}\sim N\left(0,\text{I}\sigma_{p}^{2}\right)$ and $\text{l}\text{o}\sim N\left(0,\text{I}\sigma_{lo}^{2}\right)$, respectively, where **I** is an identity matrix.

Feed intake and feed conversion data were analyzed using the following model:


$$\begin{array}{l} Y_{ijkl}=LRYS_{i}+\beta {\left(N\right)}_{j}+e_{ijk}\; \end{array}$$

Where, *Y*_*ijkl*_ is the record trait; *LRYS*_*i*_ is the effect of line-room-year-season combination; *N*_*k*_ is the number of young rabbits per cage β is the regression coefficient on this covariate and *e*_*ijk*_ is the residual effect.

A linear model was used to analyze the studied traits to test the significant differences between the levels of fixed effects and the probability of the estimated contrast being different than 0 by using the program Rabbit developed by the Institute for Animal Science and Technology (València, Spain) applying the previous model without the random effects. After some exploratory analyses, results were based on Markov chain Monte Carlo chains consisting of 60,000 iterations, with a burn-in period of 10,000, and only 1 of every 10 samples was saved for inferences.

## Results and discussion

Descriptive statistics including overall means and standard deviations of the studied traits are presented in Table 1. All the entire data was considered.

**Table 1:** Descriptive statistics [mean, standard deviation (SD), and extreme values] of the studied traits.

**Traits**	**Number**	**Mean**	**SD**	**Minimum**	**Maximum**
TB	504	8.78	2.80	1	16
TBA	504	7.88	2.96	0	14
NW	504	6.69	2.90	0	13
NM	504	5.69	2.90	0	13
BW_28_	2037	575.72	151.83	138.16	1020.16
BW_42_	1847	992.19	198.39	409.28	1690.53
BW_63_	1672	1620.46	282.86	712.70	2615.32
ADG_28−42_	2037	29.93	8.61	−8.73	53.87
ADG_42−63_	1672	29.92	5.89	8.19	58.90
ADG_28−63_	1672	29.91	6.59	5.17	55.03
DF_28−42_	340	67.75	18.25	21.14	128.39
DF_42−93_	340	121.67	21.93	28.61	214.91
DF_28−63_	340	113.44	20.58	30.51	182.96
FCR_28−63_	340	3.83	0.39	1.81	11.21

TB, total born; NBA, number born alive; NW, number weaned; NM, number marketed; BW, body weight (g); ADG, average daily gain (g/day); DF, daily feed intake (g); FCR, feed conversion ratio (g feed/ g body weight); SD, standard deviation.

### Effects of genotype and heat conditions

The observed least square means of litter and growth traits for genotypes and environmental conditions are presented in Table 2. The present results are similar to production levels previously reported for studies carried out in similar production conditions by APRI line (16–18). As expected, litter traits were negatively impacted by heat conditions, which are in agreement with most of the studies on this subject in environmental conditions. In both genotypes, TB, NBA, and NM were reduced with relevant values due to heat stress (ranged from 15 to 25%). Concerning the effects of heat conditions on the growth traits, as expected, heat stress remarkably affected all growth traits, with important reductions. The observed reduction in body weight was associated with heat-stress conditions at all ages. The reduction was 3% at weaning age, and increased with age, reaching 12 and 14% at 42 and 63 days of age, respectively. Bassuny et al. (22) reported a reduction of 7% in body weight at 28 days of age due to the increase in ambient temperatures. In addition, there was a negative effect of heat stress on the average daily gain and daily feed intake. The observed reduction in ADG ranged from 18 to 23%. Furthermore, HC environment limited the feed intake (−14% to −21%) of animals and negatively affected feed conversion (+5%) compared with NC. Similar results were obtained in previous studies (16, 23–25). The deleterious effect of heat stress on the reproductive traits in mammals is well documented (26, 27). Since rabbits are susceptible to heat stress conditions (3), lower litter traits are usually associated with an increase in ambient temperatures (28, 29).

**Table 2:** Effects of heat conditions and genotypes on the litter and growth traits^a^.

**Trait**	**Environmental conditions**		**Genotypes**	
	**Normal**	**Heat stress**	**APRI**	**NZW**
TB	9.21 ± 0.17	7.74 ± 0.21	8.89 ± 0.19	8.06 ± 0.21
NBA	8.34 ± 0.19	6.82 ± 0.26	7.88 ± 0.33	7.28 ± 0.23
NW	7.00 ± 0.20	5.95 ± 0.23	6.80 ± 0.20	6.13 ± 0.23
NM	6.18 ± 0.19	4.63 ± 0.26	5.66 ± 0.21	5.16 ± 0.24
BW_28_	586.78 ± 4.64	568.05 ± 5.48	557.76 ± 4.24	598.50 ± 5.13
BW_42_	1049.8 ± 9.95	922.1 ± 12.43	901.9 ± 9.9	1070.0 ± 9.66
BW_63_	1727.8 ± 12.79	1476.5 ± 19.34	1490.9 ± 12.86	1713.2 ± 19.04
ADG_28−42_	33.19 ± 0.48	25.30 ± 0.91	24.69 ± 0.48	33.85 ± 0.66
ADG_42−63_	32.31 ± 0.30	26.46 ± 0.69	28.06 ± 0.39	30.62 ± 0.46
ADG_28−63_	32.69 ± 0.34	25.93 ± 0.74	26.75 ± 0.62	31.91 ± 0.83
DF_28−42_	73.8 ± 0.86	58.4 ± 1.80	57.3 ± 1.16	74.9 ± 1.49
DF_42−63_	127.4 ± 1.16	109.2 ± 3.12	117.0 ± 2.06	120.6 ± 2.30
DF_28−63_	120.4 ± 1.03	100.0 ± 2.60	105.3 ± 1.58	116.9 ± 2.01
FCR_28−63_	3.72 ± 0.02	3.92 ± 0.05	3.98 ± 0.02	3.66 ± 0.03

TB, total born; NBA, number born alive; NW, number weaned; NM, number marketed; BW_28_, body weight at 28 days of age (g); BW_42_, body weight at 42 days of age (g); BW_63_, body weight at 63 days of age (g); ADG, average daily gain (g/day); DFI, daily feed intake (g); FCR, feed conversion ratio (g feed/ g body weight). ^a^The presented vales are posterior least-squares means ± SE.

Independent of environmental conditions, APRI rabbits showed important increases in TB, NBA, NW, and NM of 10, 8, 11, and 10% compared to NZW, respectively (Table 1). The superior reproductive traits of APRI rabbits could be due to the foundation of this line which was founded by the crossing between Red Baladi with the Spanish maternal line (V line) selected for litter size at weaning which has high values for litter traits (30). Moreover, after the APRI line foundation, it has been selected for litter weight at weaning (20).

Moreover, the NZW rabbits showed significantly higher body weights at all ages compared with APRI rabbits. It is worth mentioning that although the APRI line had fewer values in body weight measurements, it was superior in the reproductive traits measurements, this is mainly due to the negative phenotypic and genetic correlations between growth and reproductive traits. Ezzeroug et al. (31) reported highly negative genetic correlations between growth and litter traits in rabbits, where weaning weight was negatively correlated with litter size at birth (−0.85), number born alive (−0.92), and litter size at weaning (−0.85). Furthermore, NZW rabbits were significantly superior to the APRI line in average daily gain and daily feed intake during all periods. The average daily gain in rabbits is a very important trait in rabbit breeding, and consider one of the selection criteria in the paternal line (32, 33). Drouilhet et al. (34) reported that ADG has a high genetic correlation with BW at 63 days of age (0.81), and a low correlation with weaning weight (-0.2). Marco-Jiménez et al. (35) commented that the ambient temperature needed for rabbits is ranging from 14 to 22°C, and exceeding this range will prone rabbits to stress affecting their litter traits and the growth of young rabbits negatively. They observed significant differences between reproductive performance and body weight traits of young rabbits reared under normal conditions and those of animals under HS conditions. Within this context, Boddicker et al. (36) observed that HC during the first half of gestation may have a negative consequence on postnatal offspring growth and development. Furthermore, Habeeb (37) and Bakr (38) observed that heat conditions had a negative and significant effect on daily feed intake and body weight at weaning. Decreased feed intake results in a lower supply of nutrients, thus reducing the weight and growth rate of rabbits. This effect is in agreement with that observed by Savietto (39) and Savietto et al. (24) where heat stress reduced dry matter and digestible energy intake encountered in rabbits. Jimoh et al. (40) reported that heat conditions decreased feed intake which results in a lower supply of nutrients, thus reducing body weight, growth rate, and reproduction and increasing mortality in rabbits. Similar results, for growth traits, were obtained by Ragab et al. (41).

To evaluate the response of genotypes to environmental changes, the contrasts of each genotype under the two environmental conditions for litter traits and growth performance were calculated (Table 3). Despite their higher litter traits, APRI does showed higher reductions due to HC compared with NZW for all litter traits with relevant values, including TB (1.92 in APRI compared with 0.90 in NZW), NBA (2.22 vs. 0.54), NW (1.84 vs. 0.09), and NM (1.99 vs. 1.00), these differences indicate that the two breeds responded differently to HC, and on the whole, APRI rabbits were more sensitive to HC (the probability of contrasts being higher than 0 was 1 in the most traits) high than NWZ rabbits which seemed to be more adapted to climatic stressors. Moreover, the observed reductions in body weight were associated with HC and increased with age. The reductions were higher in NZW compared with APRI rabbits for BW at 28, 42, and 63 days of age, ADG from 28 to 42 and from 28 to 63 days of age. Similar responses for daily feed intake were observed by the two genotypes. The different responses and environmental sensitivity of genotypes were described previously by many authors (42–44), which could be due to the capacity of does to manage their body reserves usage in providing rabbits with adaptive and maintain their litter size during a stress period.

**Table 3:** Response of genotypes to environmental conditions for litter and growth traits.

**Traits**	**APRI**			**NZW**		
	**Normal vs. heat**	**HPD_95%_**	**P**	**Normal vs. heat**	**HPD_95%_**	**P**
TB	1.92	1.11, 2.75	1	0.90	0.03, 1.87	0.97
NBA	2.22	1.30, 3.05	1	0.54	−0.38, 1.58	0.86
NW	1.84	1.03, 2.75	1	0.09	−0.89, 1.01	0.58
NM	1.99	1.11, 2.85	1	1.00	−1.16, 0.91	0.97
BW_28_	4.78	−10.09, 19.57	0.72	37.59	21.07, 55.5	1
BW_42_	84.93	66.39, 102.1	1	183.18	164.2, 202.7	1
BW_63_	230.62	203.7, 255.6	1	281.67	252.6, 311.5	1
ADG_28−42_	5.74	5.00, 6.47	1	10.39	9.55, 11.26	1
ADG_42−63_	6.95	6.36, 7.52	1	4.69	4.02, 5.36	1
ADG_28−63_	6.47	5.88, 7.12	1	6.98	6.27, 7.64	1
DF_28−42_	11.11	9.51, 12.79	1	20.87	19.01, 22.74	1
DF_42−63_	19.22	17.18, 21.11	1	15.83	13.57, 18.12	1
DF_28−63_	22.6	17.65, 24.69	1	20.78	18.51, 23.1	1
FCR_28−63_	−0.23	−0.27, −0.18	1	−0.13	−0.18, −0.08	1

TB, total born; NBA, number born alive; NW, number weaned; NM, number marketed; BW, body weight (g); ADG, average daily gain (g/day); DF, daily feed intake (g); FCR, feed conversion ratio (g feed/ g body weight). HPD_95%_, Marginal Posterior highest density region covering 95% of the density; P, probability of the difference being greater than zero.

### Genotype by heat conditions interaction

Genotype by environmental conditions interaction can be observed when the difference in the performance of two genotypes depends on the environment in which the performance is measured. Therefore, G × E interaction plays an important role in the adaptation and resilience of genotypes. Table 4 shows the effects of genotype × heat conditions interaction on litter and growth traits. The presented differences between APRI and NZW genotypes under normal conditions, confirm the goodness of crossing local breeds with selected lines, such as V-line that has been selected in Spain in a climate similar to that of Egypt and used in the development of APRI line. Moreover, the obtained results indicate interaction effects on all litter traits. It can be noticed that the differences between the two lines under NC were reduced under HC, which reflect the different response of genotypes to environmental changes. Under NC, APRI litters showed relevant higher values for TB (+1.18), NBA (+1.19), NW (+1.26), and NM (+0.87) than NZW litters when reared under the same conditions, while there were relevant reductions in litter traits of APRI line in HC with significant differences (different HPD_95_ and probability of the difference between the two contrasts being greater than zero is close to 1) in NBA and NW, in which the NZW compensated for the previous differences under NC and showed higher values (0.5 and 0.48, respectively) compared with APRI litters. These results are signs of the G × H interaction and confirmed that the environmental conditions produce different physiological consequences on female rabbits, whereas the NZW breed was more flexible and less sensitive to heat stress due to the fact that NZW has been colonized and raised for many years under Egyptian conditions. The superiority of the APRI line is due to the foundation and selection process after its foundation. But, as confirmed in previous studies, does at higher production levels are more sensitive to heat stress. Marai et al. (28) reported that heat stress has negative effects on the growth of embryos, pregnancy rate, litter size, litter weight, and milk yield of does. In a similar study in rabbits, Savietto et al. (24) observed important genotype by environment interaction, where the does of the V line (which was used to develop the APRI line) showed higher litter traits than does of LP lines and the opposite occurred under heat stress conditions.

**Table 4:** Effects of genotype by heat interaction on litter and growth traits.

**Traits**	**Normal conditions**			**Heat conditions**			**Pc**
	**APRI vs. NZW**	**HPD_95%_**	**P**	**APRI vs. NZW**	**HPD_95%_**	**P**	
TB	1.18	0.47, 1.89	1	0.15	−0.92, 1.13	0.61	0.97
NBA	1.19	0.54, 1.93	1	−0.50	−1.57, 0.53	0.82	0.89
NW	1.26	0.59, 1.99	1	−0.48	−1.52, 0.53	0.82	0.79
NM	0.87	0.11, 1.65	0.99	−0.13	−1.16, 0.91	0.6	0.65
BW_28_	−50.47	−64.03, −35.16	1	−17.66	−36.51, 0.24	0.97	0.73
BW_42_	−203.9	−219.0, −186.4	1	−104.80	−126.6, −84.03	1	0.66
BW_63_	−237.6	−262.9, −215	1	−186.54	−217.9, −155.4	1	0.93
ADG_28−42_	−10.93	−11.53, −10.12	1	−6.23	−7.11, −5.31	1	0.93
ADG_42−63_	−1.63	−2.18, −1.08	1	−3.90	−4.59, −3.17	1	0.68
ADG_28−63_	−5.34	−5.92, −4.79	1	−4.83	−5.55, −4.11	1	0.70
DF_28−42_	−21.17	−22.75, −19.58	1	−11.44	−13.32, −9.36	1	0.76
DF_42−63_	−1.64	−3.51, 0.14	1	−5.03	−7.58, −2.69	1	0.71
DF_28−63_	−11.88	−13.82, −10.08	1	−10.19	−12.60, −7.75	1	0.69
FCR_28−63_	0.25	0.20, 0.29	1	0.39	0.33, 0.45	1	0.78

TB, total born; NBA, number born alive; NW, number weaned; NM, number marketed; BW, body weight (g); ADG, average daily gain (g/day); DF, daily feed intake (g); FCR, feed conversion ratio (g feed/ g body weight). HPD_95%_, Marginal Posterior highest density region covering 95% of the density; P, probability of the difference being differ than zero; Pc, probability of the difference between the two contrasts being differ than zero.

Genotype by heat condition interaction effects on growth, feed intake and feed conversion traits are presented in Table 4. Under normal conditions, NZW does had higher body weight and growth rates at different ages than APRI rabbits, these differences were significantly different than 0 to with values of 50.47 g at weaning and 237.6 g at marketing with ADG of 10.89 g/day at the first two weeks after weaning. However, these differences were changed when animals of the two lines were subjected to heat stress for BW_28_, BW_42_, BW_63_, ADG_28−42_, DF_28−42_ and DF_28−63_, while these differences were increased for ADG_42−63_, DF_42−63_ and feed conversion during the whole fattening period. As commented before, these changes of differences between the two lines, in the different environment conditions, are a good indicator for G × H, and the probability of the difference between the two contrasts being greater than zero is higher in the most traits. Therefore, it is important to take into account the environmental conditions when we will rear a line or genotype. In the same context, Sánchez et al. (45) concluded that the selection of animals should be realized under the conditions in which they will produce. Therefore, under tropical conditions, the genetic improvement of rabbits by crossbreeding between local and exotic breeds should be followed by selection in conditions similar to commercial production conditions in order to select well-adapted animals to tolerate heat stress and increase their ability to survive, grow and reproduce.

## Conclusion

Crossbreeding between local and exotic breeds helped to improve rabbits' performance. The observed results confirmed the existence of G × H interactions that affected growth and litter size traits, as different genotypes responded differently under different environmental conditions. Our results also indicated that heat stress, as a part of the environmental factors, has negatively impacted the gestation and fattening period in both genotypes, and therefore, G × H interaction should receive more attention whenever designing breeding programs.

## Data availability statement

The original contributions presented in the study are included in the article/supplementary material, further inquiries can be directed to the corresponding author.

## Ethics statement

The animal study was reviewed and approved by the Animal Care and Ethics Committee of Kafrelsheikh University, Egypt (license number, KFS1345/10).

## Author contributions

MR: formal analysis, methodology, validation, supervision, and writing—original draft. IE: conceptualization, resources, methodology, and writing. HY: writing—review and editing. MA: visualization, formal analysis, and writing—original draft. MH: methodology, validation, investigation, and writing—review and editing.

## Conflict of interest

The authors declare that the research was conducted in the absence of any commercial or financial relationships that could be construed as a potential conflict of interest.

## Publisher's note

All claims expressed in this article are solely those of the authors and do not necessarily represent those of their affiliated organizations, or those of the publisher, the editors and the reviewers. Any product that may be evaluated in this article, or claim that may be made by its manufacturer, is not guaranteed or endorsed by the publisher.
